# Thermodynamics, Charge Transfer and Practical Considerations of Solid Boosters in Redox Flow Batteries

**DOI:** 10.3390/molecules26082111

**Published:** 2021-04-07

**Authors:** Mahdi Moghaddam, Silver Sepp, Cedrik Wiberg, Antonio Bertei, Alexis Rucci, Pekka Peljo

**Affiliations:** 1 Research Group of Battery Materials and Technologies, Department of Mechanical and Materials Engineering, Faculty of Technology, University of Turku, 20014 Turun Yliopisto, Finland; mahdi.moghaddam@utu.fi (M.M.); silver.sepp@utu.fi (S.S.); cedrik.wiberg@utu.fi (C.W.); 2 Institute of Chemistry, University of Tartu, Ravila 14a, 50411 Tartu, Estonia; 3 Department of Civil and Industrial Engineering (DICI), University of Pisa, Largo Lucio Lazzarino 2, 56122 Pisa, Italy; antonio.bertei@unipi.it; 4 Department of Chemistry—Ångström Laboratory, Uppsala University, Box 538, 75121 Uppsala, Sweden; alexis.rucci@kemi.uu.se

**Keywords:** solid boosters, redox targeting, flow batteries, energy storage, redox solids

## Abstract

Solid boosters are an emerging concept for improving the performance and especially the energy storage density of the redox flow batteries, but thermodynamical and practical considerations of these systems are missing, scarce or scattered in the literature. In this paper we will formulate how these systems work from the point of view of thermodynamics. We describe possible pathways for charge transfer, estimate the overpotentials required for these reactions in realistic conditions, and illustrate the range of energy storage densities achievable considering different redox electrolyte concentrations, solid volume fractions and solid charge storage densities. Approximately 80% of charge storage capacity of the solid can be accessed if redox electrolyte and redox solid have matching redox potentials. 100 times higher active areas are required from the solid boosters in the tank to reach overpotentials of <10 mV.

## 1. Introduction

Substituting conventional carbon-based energy resources with renewables, specifically intermittent energy resources such as solar and wind, requires effective storage policies to ensure the balance between the energy production and the energy consumption [1,2]. Redox flow batteries (RFBs), as a technology in which electricity and chemical energy are interconverted by using redox-active species dissolved in electrolyte solutions stored in tanks, are proposed as a promising alternative for stationary energy storage and present advantages such as high safety, stability, flexibility, and scalability [3]. For example, RFBs are able to decouple the power from the capacity so that simply by increasing the tank size and consequently the amount of electrolyte, the storage capacity would increase with obtaining the same power output.

What hinders RFBs from gaining ground in the market of stationary energy storage is their relatively high cost and low energy density, which is limited by the solubility of the redox active species. It is possible to increase the storage capacity of RFBs by introducing redox solid storage materials to the tanks. Even systems where solid storage medium is the main contributor to energy storage could be envisaged. In this way, dissolved redox species in the electrolyte act as charge transfer mediators for the redox solid material. Therefore, while the amount of electrolyte is the same, the storage capacity of the battery will be increased and will no longer be determined solely by the solubility of the dissolved redox species. This translates to a higher energy density than that of the conventional redox flow batteries. This concept is related to semi-solid flow batteries, where redox active solids are circulated as a slurry [4]. Unfortunately, the use of suspensions of active solids needs a reformulation of the cell architecture, and these systems suffer from high viscosity and abrasive nature of the slurry and may face sedimentation issues. Solid boosted flow batteries avoid these disadvantages.

Working principle of solid boosted flow batteries can be summarized into two events occurring both in the tank and the battery cell and they are explained in depth in the next section. In the cell the electrochemical charge and discharge of the redox electrolyte red_1_/ox_1_ proceed as in a typical flow battery. In the storage tank, redox electrolyte reacts chemically with the redox active solid material ox_2_/red_2_. In this process the cell voltage is determined by the reaction in the cell while the capacity is given mostly by the solid material. Electrode materials of Li-ion batteries have a high effective concentration of charges, e.g., 22.8 M for LiFePO_4_ and 22.5 M for TiO_2_, resulting in a superior capacity. The addition of these materials in the tanks will enhance the capacity compared with the classical vanadium redox flow battery where the concentration of vanadium species is between 1.5 M and 2 M [5,6,7].

This concept, denoted as “Redox Targeting System”, was first proposed by Wang et al. [8] in 2006, and was recently reviewed by Gentil et al. [7]. In 2006 Wang et al. demonstrated the possibility of charging a target solid battery material with any dissolved redox couples in the electrolyte whose redox potential was higher than the redox potential of the solid material. Discharge was realized by a second redox couple with a lower redox potential. LiFePO_4_ was utilized as the solid material, while two osmium complexes were used as electrolytes with higher and lower redox potentials. The concept was further demonstrated in 2013 by Huang et al. [9] with LiFePO_4_ as the cathode material and in 2014 by Pan et al. [10] with Li_x_TiO_2_ as the anode material. A similar concept was developed by Wang et al. for non-aqueous lithium-ion batteries utilizing inorganic materials [4,11]. Later in 2017, Zanzola et al. reported a new lithium-free aqueous redox flow system with a similar concept denoting solid storage materials as ‘’Redox Solid Energy Boosters’’. They used polyaniline as the redox storage material in iron-containing acidic electrolytes and showed that addition of this solid storage material enhances the energy storage capacity by a factor of three, and also improves the voltage efficiency of the battery [12]. The concept of charge storage in solid boosters was described in detail with organic (2,2,6,6-Tetramethylpiperidin-1-yl)oxyl (TEMPO) derivative as redox electrolyte and copper hexacyanoferrate as the redox active solid [13]. Later in 2019, Chen et al. [6] further demonstrated the concept of redox targeting in an aqueous [Fe(CN)_6_]^4−/3−^-based electrolyte with Prussian blue utilized as the redox solid material, and reported an unprecedented volumetric capacity of 61.6 Ah/L relative to other [Fe(CN)6]^4−/3−^-based electrolytes for their redox flow battery setup. In the most recent work on the demonstration of redox targeting redox flow batteries, Vivo-Vilches et al. [14] studied the thermodynamics and the kinetics of a posolyte with LiFePO_4_/FePO_4_ as the solid material and [Fe(CN)_6_]^4−/3−^ as the redox mediator. With low current density and a very porous solid material, they achieved a near-theoretical capacity with a coulombic efficiency of 99%.

**Figure 1:**
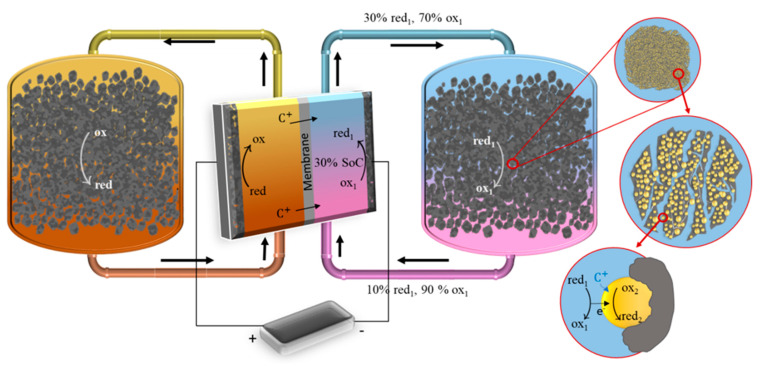
Overview of the solid booster system in a redox flow battery over multiple scales. Solid boosters are deposited in the tank as millimeter-sized porous beads, containing the redox active solid materials (ox_2_/red_2_ (yellow) for negative side), and conductive additive and binder (grey). In this example, on the negative side of the battery discharged electrolyte ox_1_/red_1_ is reduced in the cell from SoC of 10% to 30%. Reduced dissolved species will then react in the tank to reduce the solid active material ox_2_/red_2_. This reaction is typically accompanied by intercalation of a cation C^+^. Similar reactions take place at the positive side.

In the present article we formulate the thermodynamics governing these systems and illustrate how solid boosters work even when the driving forces for redox reactions are very small. We also discuss the requirements for solid boosters and redox electrolytes and discuss the engineering of booster-based flow batteries. The operating principle of a solid boosted flow battery is shown in Figure 1.

The tanks of the system described in Figure 1 contain the electrolyte solution and are filled with stationary millimeter-sized beads of redox solid materials. Beads in the tank typically consist of mainly redox active material mixed with small amounts of conductive additives such as carbon black or carbon nanotubes and binder. Each bead has a porous structure of embedded nanoparticles in contact with the carbon support, with pores from millimeters to nanometers in size. In the negative side of the battery, the redox electrolyte solution contains the ox_1_/red_1_ redox couple and the redox active material is introduced as ox_2_/red_2_ redox solid. In this paper we consider the example where initially discharged negative electrolyte solution containing 90% ox_1_ and 10% red_1_ species is pumped to the cell where ox_1_ is reduced by a cathodic current to red_1_ via reaction (i).

$${\mathrm{ox}}_{1}\left(\mathrm{aq}\right)+e^{-}\overset{\;\mathrm{cell}\;}{\to }{\mathrm{red}}_{1}\left(\mathrm{aq}\right)\;\left(i\right)$$

Based on the flow rate, the current is controlled so that the electrolyte leaves the cell at 30% state of charge (SoC), corresponding to 30% red_1_ and 70% ox_1_. Within the tank, red_1_ is oxidized back to ox_1_ in a chemical reaction with the redox active solid ox_2_ (ii)

$${\mathrm{red}}_{1}\left(\mathrm{aq}\right)+{\mathrm{ox}}_{2}\left(s\right)+C^{+}\left(\mathrm{aq}\right)\overset{\;\tan k\;}{\to }{\mathrm{ox}}_{1}\left(\mathrm{aq}\right)+{\mathrm{red}}_{2}\left(s\right)\;\left(\mathrm{ii}\right)$$

While solid species ox_2_ is reduced to red_2_, coupled with the intercalation of a cation C^+^ within the structure of the redox solid via the reaction (iii)

$${\mathrm{ox}}_{2}\left(s\right)+e^{-}+C^{+}\left(\mathrm{aq}\right)\overset{\;\mathrm{redox}\;\mathrm{solid}\;}{\to }{\mathrm{red}}_{2}\;\left(s\right)\;\left(\mathrm{iii}\right)$$

where ox_2_ is the pristine redox solid material and red_2_ is the C^+^ intercalated state. The corresponding half-reaction for the redox electrolyte is

$${\mathrm{red}}_{1}\left(\mathrm{aq}\right)\overset{\;\tan k\;}{\to }{\mathrm{ox}}_{1}\left(\mathrm{aq}\right)+e^{-}\;\left(\mathrm{iv}\right)$$

ox_1_ can then once again be reduced in the cell. The charging continues until an electrochemical equilibrium is reached between the electrolyte and the redox solid. Only the negative electrolyte is considered in this treatment, but an analogous process would take place on the positive side.

## 2. Result and Discussion

### 2.1. Thermodynamics of Solid Boosters

#### 2.1.1. Thermodynamics of Solid Boosters: Equilibrium

In this section we consider the charge transfer reactions between solid and liquid species in equilibrium, for the negative side. Firstly, the redox active species in solution is reduced at the electrode in the cell to form red_1_ from ox_1_ according to reaction (v) with the corresponding Nernstian relationship in Equation (1).

$$\underset{\mathrm{cell}}{\underbrace{{\mathrm{ox}}_{1}\left(\mathrm{aq}\right)+e^{-}⇌{\mathrm{red}}_{1}\left(\mathrm{aq}\right)}}\;\left(v\right)$$

(1) $$E_{1}=E_{1}^{0}+\frac{RT}{F}ln\left(\frac{a_{ox_{1}}}{a_{red_{1}}}\right)$$

where a is the activity of the indexed species. The consecutive half-cell charging reaction of the redox solid is shown in reaction (vi) and Equation (2).

$${\mathrm{ox}}_{2}\left(s\right)+C^{+}\left(\mathrm{aq}\right)+e^{-}⇌{\mathrm{red}}_{2}\left(s\right)\;\left(\mathrm{vi}\right)$$

(2) $$E_{2}=E_{2}^{0}+\frac{RT}{F}ln\left(\frac{a_{ox_{2}}a_{C^{+}}}{a_{red_{2}}}\right)$$

The overall reaction (vii), has a potential expression, Equation (3), that corresponds to the potential difference between reactions (v) and (vi).

$${\mathrm{red}}_{1}\left(\mathrm{aq}\right)+{\mathrm{ox}}_{2}\left(s\right)+C^{+}\left(\mathrm{aq}\right)⇌{\mathrm{ox}}_{1}\left(\mathrm{aq}\right)+{\mathrm{red}}_{2}\left(s\right)\;\left(\mathrm{vii}\right)$$

(3) $$E_{overall}=E_{2}^{0}-E_{1}^{0}+\frac{RT}{F}ln\left(\frac{a_{ox_{2}}a_{C^{+}}}{a_{red_{2}}}\right)\;-\frac{RT}{F}ln\left(\frac{a_{ox_{1}}}{a_{red_{1}}}\right)\;=E_{2}^{0}-E_{1}^{0}+\frac{RT}{F}ln\left(\frac{a_{ox_{2}}a_{red_{1}}a_{C^{+}}}{a_{ox_{1}}a_{red_{2}}}\right)\;$$

The driving force for the charging of the redox solid is thus the Nernst potential difference E_2_-E_1_ and depends on the standard potentials for the redox couples of the redox-active materials in the solution and booster. As the cation is intercalated in the redox solid upon reduction, its activity is also included in the potential expression. The concentration of the cation will vary with the state of charge, and therefore needs to be chosen judiciously, taking the amount of redox solid into account. However, if solid boosters are used on both the positive and negative side, a rocking-chair effect where the cation migrates from one side to the other is seen, and the problem is obviated. The activity of the solid species cannot be set to unity and removed from the expressions, this would lead to a completely horizontal charging curve, and this is not observed experimentally. However, a discussion of activities is considered out of the scope of this article and is given elsewhere [15]. Shortly, activities of solids are considered to vary between 0 and 1, depending on the molar fractions of the ox_2_ and red_2_.

The same considerations apply to the positive side of the battery upon charge, with the only exception that electrochemical reactions take place in the opposite direction, that is, the electrolyte is reduced at the solid booster particle and the solid booster undergoes oxidation. Thus, Equations (1)–(3) hold for the positive side too, although the driving force for charging at the positive side is *E_overall_* = *E*_2_ − *E*_1_ < 0 or, equivalently, *E*_1_ > *E*_2_, which is the opposite sign of the driving force considered for the negative side, i.e., *E_overall_* = *E*_2_ − *E*_1_ > 0 or *E*_2_ > *E*_1_ as considered above.

For the sake of discussion, let us assume that the activity for the cation is unity and the state of charge varies linearly with the activities of the redox species. Considering the positive side of the battery, the state of charge (SoC) for the dissolved species (ox_1_/red_1_) and solid booster (ox_2_/red_2_) respectively can be defined as follows:

(4) $$SoC_{1}=a_{{\mathrm{ox}}_{1}}=1-a_{{\mathrm{red}}_{1}}$$

(5) $$SoC_{2}=a_{{\mathrm{ox}}_{2}}=1-a_{{\mathrm{red}}_{2}}$$

and substitution into Equation (3) gives

(6) $$E_{overall}=E_{2}^{0}-E_{1}^{0}+\frac{RT}{F}\ln\left(\frac{SoC_{2}\left(1-SoC_{1}\right)}{\left(1-SoC_{2}\right)SoC_{1}}\right)\;$$

At equilibrium, $E_{overall}=0,$ and Equation (6) can be reorganized into

(7) $$SOC_{2}=\frac{SOC_{1}e^{\frac{F\left(E_{1}^{0}-E_{2}^{0}\right)}{RT}}}{\left(1-SOC_{1}\right)+SOC_{1}e^{\frac{F\left(E_{1}^{0}-E_{2}^{0}\right)}{RT}}}$$

**Figure 2:**
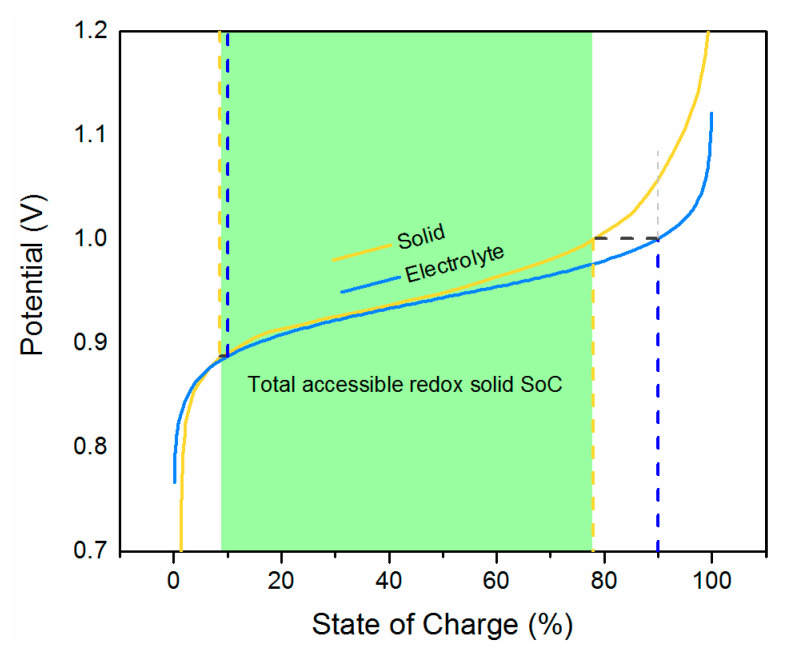
Accessible SoC of solid booster as a function of electrolyte potential with matching standard potentials.

As an example, we take the copper hexacyanoferrate (CuHCF) as a solid booster in the positive side of the system. Experimental potentials as a function of state of charge for the solid from reference [16] are shown as the yellow line in Figure 2. In the same graph, potentials according to the Nernst equation for a dissolved redox couple with a closely matching equilibrium potential is shown. The experimental curve for the solid material deviates from the Nernstian behavior due to changes in the activities of the solid species [15]. In this scenario, it is assumed that the electrolyte is only cycled between 10% and 90% SoC, depicted by the blue vertical dashed lines.

Looking at Figure 2, the potential for the electrolyte, *E*_1_, at 90% SoC equals the potential for the solid material, *E*_2_, at about 78% SoC, signifying the maximum possible depth of charge of the solid booster in this system. Analogously, *E*_1_ at 10% SoC corresponds to approximately 8% SoC in the solid material. Consequently, in this scenario, 68% of the booster capacity is accessed. If the flow battery could be operated at SoC interval between 5 and 95%, a slightly higher booster capacity would be accessible. Typically, commercial vanadium flow batteries operate at SoC limits of 5 to 85% [17], mostly to avoid precipitation of V_2_O_5_, so a SoC range of 5 and 95% could be feasible. Wider SoC ranges are not practical due to the mass transfer limits decreasing the accessible charging and discharging powers.

**Figure 3:**
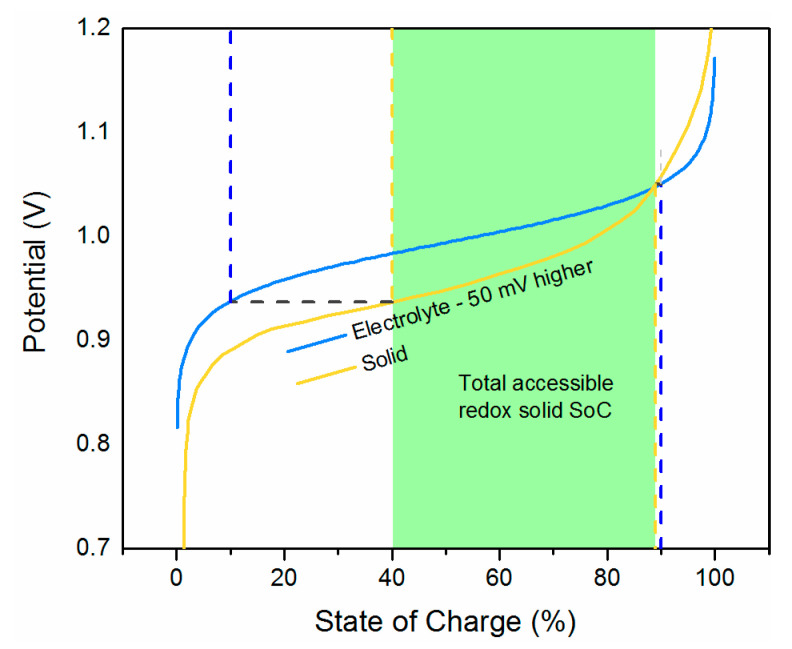
Accessible SoC of solid booster as a function of electrolyte potential. $E_{1}^{0}$ is 50 mV higher than $E_{2}^{0}$.

The case where the equilibrium potential of the electrolyte, $E_{1}^{0}$, is shifted 50 mV higher than that of the solid, $E_{2}^{0}$, is shown in Figure 3. The accessible SoC of the solid during charging is increased to close to 90%, but during discharge, only approximately 40% can be reached. The total accessible SoC of the solid booster reaches around 50% and is thus heavily limited by the disparity of potentials between the redox couples in the electrolyte and solid.

**Figure 4:**
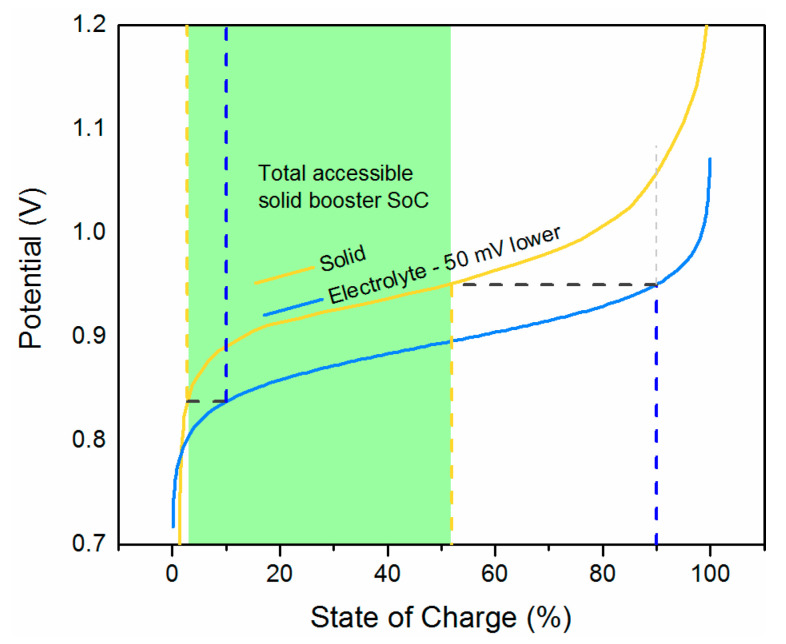
Accessible SoC of solid booster as a function of electrolyte potential. $E_{1}^{0}$ is 50 mV lower than $E_{2}^{0}$.

Similarly, the situation if $E_{1}^{0}$ is 50 mV lower than $E_{2}^{0}$ is shown in Figure 4. Here, *E*_1_ at 90% SoC allows the electrolyte to equilibrate with the solid up to approximately only 52%, showing a comparable capacity limitation to the previous example.

**Figure 5:**
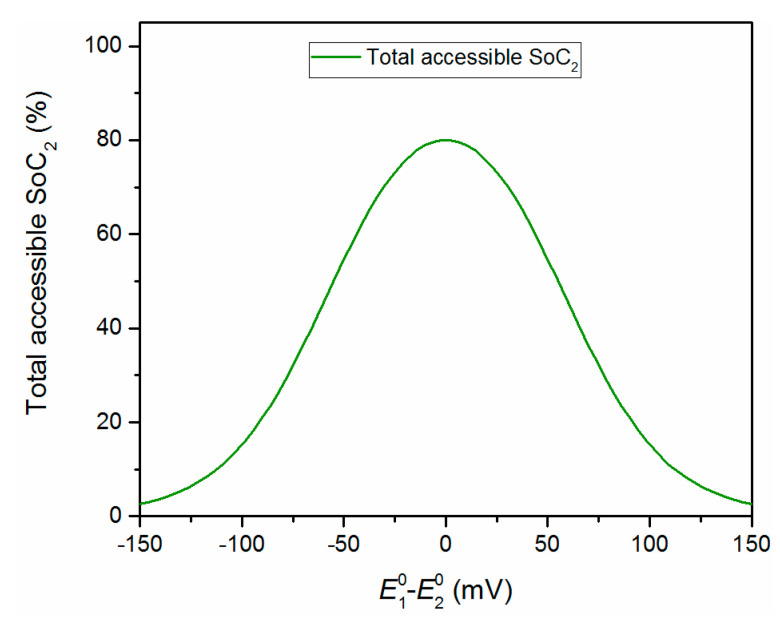
SoC_2_ as a function of potential mismatch between $E_{1}^{0}$ and $E_{2}^{0}$.

For a Nernstian system, based on Equation (7), the potential mismatch between $E_{1}^{0}$ and $E_{2}^{0}$ is related to the accessible SoC of the solid booster in Figure 5. There, it is seen that the full 80%, which corresponds to the limits of SoC_1_, is accessed when the equilibrium potentials agree. However, a seemingly small mismatch of 50 mV in either direction decreases SoC_2_ by 30%.

One way to circumvent the limited accessed solid booster capacity from mismatching potentials is to use two different dissolved redox-active species with respective potentials higher and lower than that of the solid booster [8,9,10]. This strategy was illustrated in many initial systems, where for example solid FePO_4_ was charged with the dibromoferrocene (FcBr_2_/FcBr_2_^+^) redox couple and discharged with the ferrocene (Fc/Fc^+^) redox couple. In this case, the same reaction scheme, i.e., (v)–(vii), will take place during charging. However, when discharging, the booster will reduce the second dissolved redox-active material, ox_3_ to form red_3_, instead:

$${\mathrm{red}}_{2}\left(s\right)+{\mathrm{ox}}_{3}\left(\mathrm{aq}\right)⇌{\mathrm{red}}_{3}\left(\mathrm{aq}\right)+{\mathrm{ox}}_{2}\left(s\right)+C^{+}\left(\mathrm{aq}\right)\;\left(\mathrm{viii}\right)$$

red_3_ is in turn oxidized in the cell, completing the charge/discharge cycle.

$${\mathrm{red}}_{3}\left(\mathrm{aq}\right)⇌{\mathrm{ox}}_{3}\left(\mathrm{aq}\right)+e^{-}\;\left(\mathrm{ix}\right)$$

(8) $$E_{3}=E_{3}^{0}+\frac{RT}{F}\ln\left(\frac{a_{{\mathrm{ox}}_{3}}}{a_{{\mathrm{red}}_{3}}}\right)$$

and the discharging voltage is thus given by the following relationship:

(9) $$E_{{overall}^{\prime }}=E_{3}^{0}-E_{2}^{0}+\frac{RT}{F}\ln\left(\frac{a_{{\mathrm{ox}}_{3}}a_{{\mathrm{red}}_{2}}}{a_{{\mathrm{ox}}_{2}}a_{{\mathrm{red}}_{3}}a_{C^{+}}}\right)$$

This setup allows for a large utilization of the solid booster capacity but has the drawback that the battery is charged using a reaction with a higher potential than it is discharged with. In order to exemplify the energetics of having a three-redox-couple-system, the following scenario is considered: absence of overpotentials, system in equilibrium (all activities are unity) and the nominal cell voltage is *E_f_* (for example the difference in potential between the anodic half-cell and $E_{2}^{0}$). The voltage efficiency, *VE*, is then:

(10) $$VE=\frac{E_{f}+E_{overall’}}{E_{f}+E_{overall}}=\frac{1+E_{3}^{0}-E_{2}^{0}}{1+E_{2}^{0}-E_{1}^{0}}$$

Assuming for simplicity $\Delta E=-(E_{3}^{0}-E_{2}^{0})=E_{2}^{0}-E_{1}^{0}$, ΔE  is related to the *VE*:

(11) $$VE=\frac{E_{f}-\Delta E}{E_{f}+\Delta E}$$

yielding Figure 6.

CuHCF has an SoC curve with quite close to ideal behavior, but other solid materials such as LiFePO_4_ with steeper SoC curves, or materials such as LiMn_2_O_4_ with two plateaus need to be treated separately. For such systems, the accessible SoC range will be smaller. Therefore, it is crucial to perform this analysis based on experimental SoC curves for the redox solid materials.

#### 2.1.2. Thermodynamics of Solid Boosters: Dynamics

**Figure 7:**
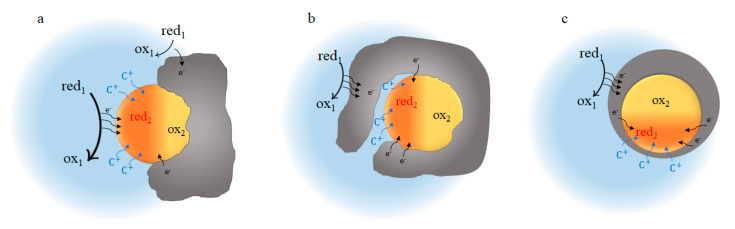
Possible pathways for charge transfer between redox electrolyte red_1_/ox_1_ and redox solid red_2_/ox_2_: (**a**) Direct chemical charge transfer; (**b**) Electron transfer through conductive additive; (**c**) Charge transfer in an enclosed system, where also the cation has to go through a carbon material.

After considering the equilibrium thermodynamics of solid boosters, the next question is related to dynamics. In this section, we illustrate the different possible pathways (Figure 7) for the charge transfer reactions between redox electrolyte and redox solids in the negative side of the system in Figure 1.

Redox solid nanoparticles are in contact with the conductive carbon support and electrons can easily be supplied for the particles from the carbon. Therefore, the active nanoparticles for energy storage will be those that are accessible to C^+^ species. The cation can reach the nanoparticle via the electrolyte-nanoparticle interface (Figure 7a,b) or through the carbon support in low-thickness regions (nanometer scale) [18] (Figure 7c).

If the redox solid nanoparticle has access to both the C^+^ and the red_1_ species in the solution (Figure 7a), reaction (iv) can take place directly at the electrolyte-nanoparticle interface and electrons enter the redox solid via a solution-solid heterogeneous transfer. At the same time, C^+^ species are intercalated into the redox solid nanoparticle’s structure via the reaction (iii). Alternatively, conductive additives could mediate the electron transfer. In this case, electron transfer from electrolyte to carbon could take place anywhere, and the conductive additive shuttles the electrons to the solid particle. This pathway is called redox electrocatalysis. The actual mechanism (chemical reaction) of redox electrocatalysis depends on which steps are rate-limiting.

The red_1_ species arriving from the cell by convection may not be able to enter into all the pores within the beads by diffusion, i.e., there will be a concentration gradient of the red_1_ species inside the bead. For the redox solid nanoparticle that is in contact with the electrolyte but is located deep enough within the beads, the scenario would be different (Figure 7b). Here, reaction (iv) takes place on the carbon support and the resulting electrons are then shuttled via the carbon to the redox solid. Instead of a solution-solid heterogeneous electron transfer, electrons transfer through a solid-solid interface from the carbon to the redox solid nanoparticle. We assume that the concentration of cation C^+^ in the electrolyte is sufficient so that it does not limit the intercalation reaction. The general intercalation reaction would be again the reaction (iii) but with one difference, that the electrons are supplied through a different pathway (from the carbon support rather than the electrolyte-redox solid interface).

For the case where C^+^ travels through a thin carbon layer to reach the nanoparticle (Figure 7c), the situation is similar to the latter case (Figure 7b).

### 2.2. Charge Storage and Kinetics of Redox Solid Materials

The kinetics of redox solid flow batteries can be divided into two main sections: the cell and the tank. In the cell, the main kinetic parameters, which define the power density of the battery, relate to the transport of the redox couple to the electrode surface in the cell and the electron transfer between the solution and the electrode. Therefore, the mechanisms in the cell are well-known [19]. Meanwhile, only very few works have studied the kinetics of charge storage in the redox solid materials with redox targeting within the tank [13,14,20,21,22,23,24]. In an attempt, Li_x_FePO_4_ (0 ≤ x ≤ 1) was coated on a double-layer electrode with an insulating Al_2_O_3_ layer and was charged and discharged with the Fc and FcBr_2_^+^ redox couple with a biased electrode [20]. Apparent rate constants in the range of 2.2 × 10^−6^ to 4.4 × 10^−6^ cm/s for uncoated Li_x_FePO_4_ and 4 to 6 times larger ones for carbon coated Li_x_FePO_4_ were reported based on measuring the length that redox species diffused into the Li_x_FePO_4_ structure. It was assumed that the reaction rate was limited by transport of the charge carriers in the solid with no more specifications. Elsewhere [21], the same redox solid and redox mediator system without any carbon coating was studied with scanning electrochemical microscopy (SECM) and effective rate constants for the lithiation and the delithiation processes were reported as 3.70 × 10^−3^ cm/s and 6.57 × 10^−3^ cm/s, respectively. With this approach, an interfacial rate constant for the intercalation reaction, valid for an interaction volume of 1–3 nm inside the active material, was reported and this made the measurement independent of diffusion of Li^+^ within the deeper regions of the redox solid. Therefore, 3 orders of magnitude higher rate constants compared to that of previous study could be explained [20].

An ample amount of studies has been done on the lithium intercalation reaction in LiFePO_4_ with direct biasing of the solid material instead of employing redox targeting, and some of them are useful to extend our knowledge of coupled ion-electron transfer (CIET) in redox targeting of redox solid materials. Bai et al. [25] showed that in charging and discharging of micrometer scale carbon coated LiFePO_4_ (LiFePO_4_:carbon:binder = 8:1:1) with a porous structure, the charge transfer reaction rate is limited by electron transfer at the carbon-LiFePO_4_ (solid-solid) interface. This result was contradictory with the conventional assumption that the rate is limited by the diffusion of Li^+^ in LiFePO_4_ structure. They showed that the Butler-Volmer (BV) kinetics model only focuses on Li^+^ in predicting the intercalation behavior and neglects the importance of the electron transfer; thus wrongly detecting the rate-limiting step. They proved that the Marcus–Hush–Chidsey (MHC) kinetics model could precisely predict the mechanism of intercalation with decoupling the effects of the ion transfer and the electron transfer, and therefore could detect the solid-solid interface electron transfer as the rate-limiting step. Elsewhere [26], they introduced a general theory for CIET kinetics, which is the case under study here, and exhibited its accurate prediction of reaction rates and rate-limiting steps for LiFePO_4_. Their general formula reduces to other kinetics models under some conditions. For moderate overpotentials and for two extreme conditions that either the ion transfer or the electron transfer is the rate-limiting step, the formula reduces to the Butler-Volmer kinetics model. Therefore, it is noteworthy to mention that although the BV kinetics model cannot detect the rate-limiting step in a CIET reaction, it can be still employed for definition of the charge transfer current, but of course with maintaining the aforementioned extreme conditions.

Here, we try to bring light on the phenomenon of storing the charge within the redox solid material based on Fermi level equilibration. So far, we have demonstrated the concept of Fermi level equilibration for some other systems [27,28,29]. Here, we utilize this concept to explain the charge transfer between the redox electrolyte and the redox solid nanoparticles, via the carbon support.

The Fermi level of an electron is a level of energy where the probability of finding an electron is equal to 1/2. For an electron in a redox couple in the electrolyte, the Fermi level is the electrochemical potential of the electron in that redox couple and is equal to the work of bringing an electron from vacuum (where the energy is by definition 0) to the redox couple in the electrolyte. This electrochemical potential is almost always negative, i.e., the reaction to bring an electron from vacuum to any system is spontaneous. For further information on the Fermi level of an electron on a redox couple in the solution, see our previous works [27,30] as well as the excellent review by Reiss [31]. The Fermi level of electrons in the solution is linearly dependent on the Nernst potential of the redox couple in solution, according to Equation (12).

(12) EF=−e[Eox/red+ϕw+[EH+/12H20]AVS]

where e is elementary charge, $E_{\mathrm{ox}/\mathrm{red}}$ is the Nernst potential of the redox electrolyte, [EH+/12H20]AVS = 4.44 V is the potential of the standard hydrogen electrode (SHE) on the absolute vacuum scale and $\phi_{w}$ is the Galvani potential (also called the inner potential) of the aqueous phase.

Most of the redox solid materials, including those used as active materials in lithium-ion batteries, have a poor conductivity, needing a conductive additive for the electronic conduction. These materials can be considered as insulators. The Fermi level of an electron in the body of an insulator redox solid is equal to the required work to bring one electron to the redox solid body from vacuum, analogously to the Fermi level in a redox electrolyte.

**Figure 8:**
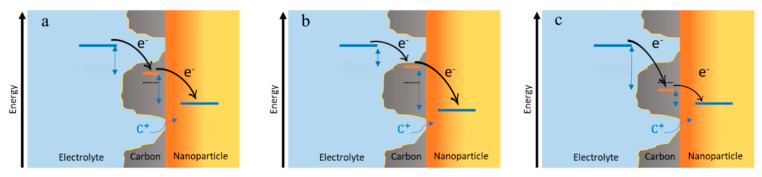
Fermi level equilibration of the carbon between redox electrolyte red_1_/ox_1_ and redox solid red_2_/ox_2_ nanoparticle. The position of the Fermi level of the carbon depends on the rates of the half-reactions taking place at its two ends and locates: (**a**) right in the middle of Fermi levels of the redox electrolyte and the redox solid when the product of exchange current densities and surface areas of both half-reactions are equal ($A_{\mathrm{ox}}i_{0,\mathrm{ox}}=A_{\mathrm{red}}i_{0,\mathrm{red}}$); (**b**) closer to the Fermi level of the oxidation half-reaction when $A_{\mathrm{ox}}i_{0,\mathrm{ox}}\gg A_{\mathrm{red}}i_{0,\mathrm{red}}$; (**c**) closer to the Fermi level of the reduction half-reaction when $A_{\mathrm{ox}}i_{0,\mathrm{ox}}\ll A_{\mathrm{red}}i_{0,\mathrm{red}}$ (orange indicates the intercalated zone in nanoparticle, and yellow indicates the pristine nanoparticle).

Carbon functions as an electron mediator and shuttles the electron between the electrolyte and the nanoparticle. Therefore, the Fermi level of the carbon support locates somewhere between the Fermi levels of the electrolyte, and the Fermi level of the nanoparticle (Figure 8).

The position of the Fermi level of the carbon depends on the rates of the reactions taking place at its two ends. Where the carbon comes into contact with the electrolyte, a conventional heterogeneous electron transfer occurs, which follows the BV kinetics model. If the overpotentials are small, Butler-Volmer equation can be linearized, and the current for the oxidation half-reaction ${\mathrm{red}}_{1}\overset{\;}{\to }{\mathrm{ox}}_{1}+e^{-}$, when neglecting the concentration polarization can be expressed as

(13) $$I_{ox}=A_{ox}i_{0,ox}f\eta_{ox}$$

where A is the area available for the reaction, *i*_0_ is the exchange current density, f=F/RT, and η is the overpotential which is the driving force for the electron to move from one Fermi level to another one.

At the other end of the carbon, the intercalation reaction takes place, which is a CIET. In the CIET, a cation enters the redox solid structure while concertedly, in order to maintain charge neutrality, an electron travels to the cation. This simultaneity complicates the kinetics of this CIET. In here, we assume that the current for the reduction half-reaction ${\mathrm{ox}}_{2}+e^{-}+C^{+}\overset{\;}{\to }{\mathrm{red}}_{2}$ can be written in terms of linearized BV kinetics for small overpotential as

(14) $$I_{\mathrm{red}}=A_{\mathrm{red}}i_{0,\mathrm{red}}f\eta_{\mathrm{red}}$$

In this situation, the carbon experiences a mixed potential, and the oxidation half-reaction and the reduction half-reaction proceed at the same overall reaction rate (*I*_ox_ = *I*_red_). Let us gather together the product of exchange current density and area as *I*_0,ox_ = *A*_ox_*i*_0,ox_ and *I*_0,red_ = *A*_red_*i*_0,red_, which are the effective exchange currents of the half-reactions. We assume that cation transfer does not limit the reaction. To maintain the equality of the reactions’ current, the only modifiable parameters are the overpotentials and exchange current densities that depend on concentrations. The overpotential is the driving force for the electron to move from one Fermi level to another one and is schematically equal to the vertical distance of the Fermi levels in different media (Figure 8). If *I*_0,ox_ = *I*_0,red_, both half reactions need a similar driving force to maintain an equal current (*I*_ox_ = *I*_red_), and therefore, the Fermi level of the carbon locates in a quasi-steady state right in the middle of the Fermi level of the electrolyte and the Fermi level of the nanoparticle (Figure 8a).

If the exchange currents *I*_0,ox_ and *I*_0,red_ differ considerably in magnitude, either because of different areas (*A*_ox_ ≠ *A*_red_) or different exchange current densities (*i*_0,ox_ ≠ *i*_0,red_), the Fermi level of the carbon locates in a quasi-steady state position closer to the Fermi level of the side with the larger exchange current density (the faster reaction) (Figure 8b,c). In this way, the slower reaction will experience a larger driving force than that of the faster one and both half reactions can proceed with the same reaction rate (*I*_ox_ = *I*_red_). In principle, should the exchange current densities differ (*i*_0,ox_ ≠ *i*_0,red_), one might tailor the areas in order to ensure comparable driving forces $\eta_{\mathrm{ox}}\approx \eta_{\mathrm{red}}$ (i.e., $A_{\mathrm{ox}}/A_{\mathrm{red}}\approx i_{0,\mathrm{red}}/i_{0,\mathrm{ox}}$) in order to avoid excessive electrochemical losses for one of the half-reactions. For more detailed analysis, full Butler-Volmer equations considering mass transport should be evaluated, requiring numerical simulations.

**Figure 9:**
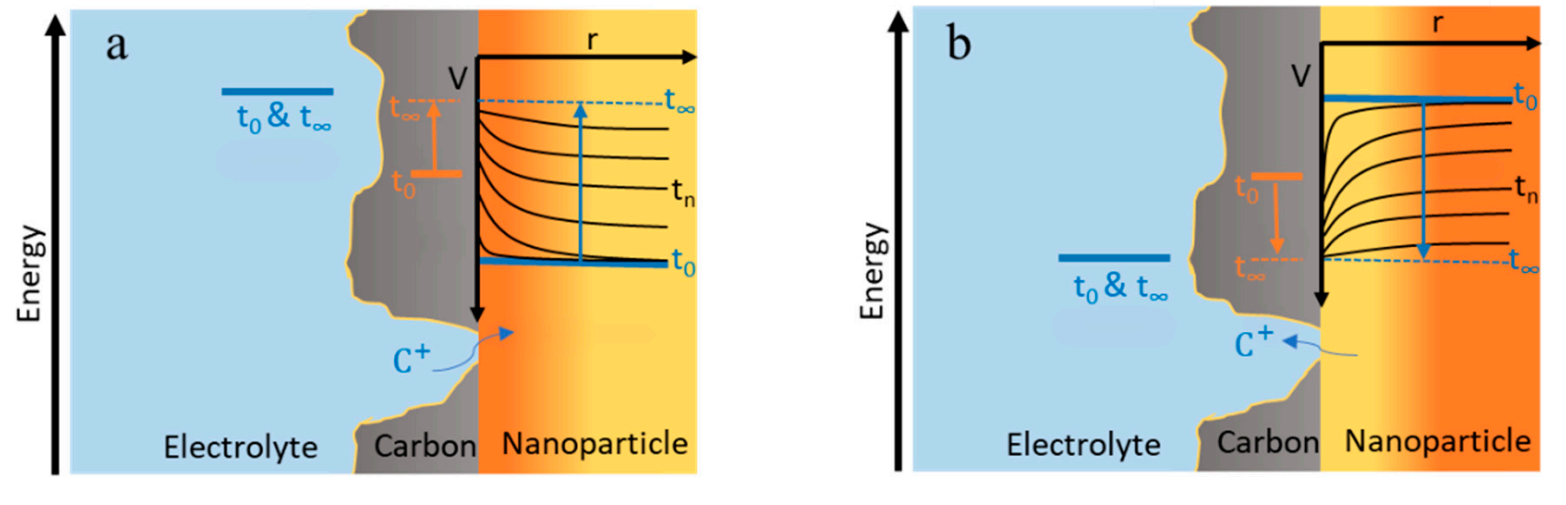
Equilibration of the Fermi level of the redox solid nanoparticle with a diffusion controlled profile during: (**a**) charging the nanoparticle when it shifts from a lower initial energy level than that of the electrolyte (*t*_0_) to the fixed Fermi level of the electrolyte at equilibrium (*t*_∞_); (**b**) discharging the nanoparticle when it lowers from a higher initial energy level (*t*_0_) to the fixed Fermi level of the electrolyte at equilibrium (*t*_∞_). The Fermi level of the carbon always locates between the Fermi level of the electrolyte and the redox solid. At equilibrium (*t*_∞_), all Fermi levels are located at the fixed Fermi level of the electrolyte.

Now we consider the hypothetical situation (Figure 1) where the SoC of the electrolyte arriving in the tank (30% red_1_) is fixed by the electrochemical reaction in the cell (SoC = 30%). The Fermi level of the electrolyte remains in a steady state equal to the potential dictated by the SoC. Charged species of the redox electrolyte deliver the charge to the redox solid nanoparticle. As shown in Figure 9a, at *t*_0_ the charging of the battery starts and the partially charged electrolyte with 30% red_1_ and 70% ox_1_ is pumped to the tank. The Fermi level of the redox solid material, independently of the distance from its surface, is located at a lower energy level compared to that of the electrolyte. When the electrolyte reaches the redox solid nanoparticle, chemical charging begins, and the Fermi level at the surface of the redox solid shifts to a higher energy level. The SoC of the electrolyte entering the cell is considered constant during charging. For deeper regions of the solid, e^−^-C^+^ pair must diffuse into the solid and thus the increase of the Fermi level within the solid will be diffusion controlled. The gradient of the Fermi level at the surface of the redox solid reaches its maximum at the start of the charging process of the redox solid. As the charging of the redox solid nanoparticle continues, its Fermi level gradually equilibrates with the Fermi level of the electrolyte with a diffusion-controlled profile. The Fermi level of the carbon also shifts to higher energy levels but always locates between the Fermi level of the electrolyte and the Fermi level of the redox solid material in order to balance the driving forces for the electron transfer reactions. The potential of the carbon can be considered floating. At equilibrium (*t*_∞_), the Fermi level of the solid material and the carbon will reach the Fermi level of the electrolyte and all Fermi levels will be equal. 

As time passes, the battery will become charged and the Fermi level of the electrolyte entering the cell will start to change. For discharging, as shown in Figure 9b, the scenario is reversed and at *t*_0_ the Fermi level of the electrolyte is fixed at a lower energy level than that of the charged redox solid. At *t*_n_, the cation transfers to the electrolyte and the Fermi level of the redox solid with a diffusion-controlled profile shifts to the lower energy levels. At equilibrium (*t*_∞_), the Fermi level of the redox solid material and the carbon will reach the fixed Fermi level of the electrolyte.

### 2.3. System Design and Techno-Economic Considerations

The next question to address is how fast the charge shuttling by the redox mediators can be. In here, we assume that the rate-determining step is the electron transfer reaction (iv) from the redox electrolyte to the redox solid. This is the simplification, assuming that all the overpotential for the reaction would be on the reaction (iv). In reality, the overpotential available for this reaction lies somewhere between the Fermi levels of the electrolyte and the solid. The available surface area for reaction (ii) per volume of the tank (*A*_tank_/*V*_tank_) is equal to the available surface area for reaction (i) per volume of the anode compartment of the cell (*A*_cell_/*V*_cell_).

With assuming large enough overpotentials, backward reactions of reaction (i) and reaction (ii) become negligible. Hence, in this scenario respecting the Butler-Volmer equation with Equation (15), currents corresponding to reaction (i) and (ii) can be expressed as *I_cell_* with Equation (16) and *I_tank_* with Equation (17) respectively.

(15) $$I=Ai_{0}(e^{\alpha f\eta }-e^{\left(\alpha -1\right)f\eta })$$

(16) $$I_{\mathrm{cell}}=-A_{\mathrm{cell}}i_{0\mathrm{cell}}e^{\left(\alpha -1\right)f\eta_{\mathrm{cell}}}$$

(17) $$I_{\tan k}=A_{\tan k}i_{0\tan k}e^{\alpha f\eta_{\tan k}}$$

where $\eta_{\mathrm{cell}}<0$ and $I_{\mathrm{cell}}<0$ while $\eta_{\tan k}>0$ and $I_{\tan k}>0$. $i_{0}$ is the exchange current density for the electron transfer between the ox_1_/red_1_ redox couple and the carbon. It depends strongly on the concentration, physical properties of the electrode and the thermodynamic properties of the system in which the reaction takes place [19]. For simplicity α, the electron transfer coefficient, is assumed to be 0.5 in Equations (16) and (17).

If we assume that the cathodic current in the cell, *I*_cell_, which is the flow of the electrons from the electrode to ox_1_ species, is as large as the flow of electrons from red_1_ species to the solid in the tank, *I*_tank_, the situation can be expressed as

(18) $$-I_{\mathrm{cell}}=I_{\tan k}\Rightarrow A_{\mathrm{cell}}i_{0}e^{\left(\alpha -1\right)f\eta_{\mathrm{cell}}}=A_{\tan k}i_{0}e^{\alpha f\eta_{\tan k}}$$

and rearranging it gives

(19) $$\frac{A_{\mathrm{cell}}i_{0,\mathrm{cell}}}{A_{\tan k}i_{0,\tan k}}=\frac{e^{\alpha f\eta_{\tan k}}}{e^{\left(\alpha -1\right)f\eta_{\mathrm{cell}}}}$$

For a reversible reaction α is generally estimated as 0.5 [32] and therefore from Equation (19) $\eta_{t\mathrm{ank}}$ can be expressed as:

(20) $$\eta_{\tan k}=\left|\eta_{\mathrm{cell}\;}\right|+\frac{ln\;\left(\frac{A_{\mathrm{cell}}i_{0,\mathrm{cell}}}{A_{\tan k}i_{0,\tan k}}\right)}{0.5\;f}$$

This expression is valid when overpotentials are large, typically more than 60 mV. More realistic scenario is to assume that the overpotential is small for the reaction in the tank. In this case, the Butler-Volmer Equation (15) can be linearized for the tank, while reverse reaction can be neglected for the cell. The final outcome is:

(21) $$-I_{\mathrm{cell}}=I_{\tan k}\Rightarrow A_{\mathrm{cell}}i_{0}e^{\left(\alpha -1\right)f\eta_{\mathrm{cell}}}=A_{\tan k}i_{0}f\eta_{\tan k}$$

(22) $$\eta_{\tan k}=\frac{A_{\mathrm{cell}}i_{0,\mathrm{cell}}e^{\left(1-\alpha \right)f\left|\eta_{\mathrm{cell}}\right|}}{A_{\tan k}i_{0,\tan k}f}$$

The electrochemical reaction of dissolved redox couples occurs in the electrodes of RFBs. Ideally, the electrodes do not take part in the redox reactions, but provide the active surface. The main properties of good electrode material are excellent electrical conductivity, high specific surface area, stability in the applied operating potential range of the RFB, and chemical inertness against electrolytes. Therefore, a good option for electrode material is high-surface-area carbon felt [33].

The chemical reaction between the dissolved redox active material and the redox solid material occurs in the solid booster beads in the tank of the RFB. These beads should have similar properties as mentioned for the electrode material above. High specific surface area and electrical conductivity can be achieved using carbon black, carbon nanotubes or similar materials. The solid active material itself should be insoluble in the solution used in the RFB. Thirdly, a binder material is needed to attach the solid active material and conductive additive together and form a solid booster bead. The binder material must not decrease the specific surface area and electron conductivity of the bead too much. The binder material is also responsible for the physical stability of the bead in the process of charging and discharging the RFB.

A high specific surface area of the solid beads is according to the BV equation one of the most important factors increasing the driving force for the electrochemical reactions in the electrodes. In case of discharging, the chemical reaction in a solid booster bead is the driving force for the electrochemical reaction in the electrode. Equations (20) and (22) can be used to estimate what kinds of *A*_tank_/*A*_cell_ ratios are required to so that the current values for the tank and cell are equal (*I*_tank_ = |*I*_cell_|).

In the example of phosphonate group−substituted viologen | ferrocyanide flow battery [34] the data are following: at 90% SOC and at the current density 300 mA/cm^2^ the high frequency resistance (*R*_el_) of the cell is 1.3 Ω cm^2^. This current corresponds to ca. 10% SoC change, i.e., the Nernst potential of the electrolyte would change by 21 mV. Making the estimation that voltage drop in polarization curve (*V*_OCV_-*V_i_*) is sum of *iR*-drop and of two equal overpotentials (for cathode and anode reaction), the calculated overpotential for one electrode (|*η*_cell_|) in the cell is 0.055 V (Equation (23)).

(23) $$\left|\eta_{\mathrm{cell}}\right|=\left(V_{\mathrm{OCV}}-V_{i}-iR_{\mathrm{el}}\right)/2$$

**Figure 10:**
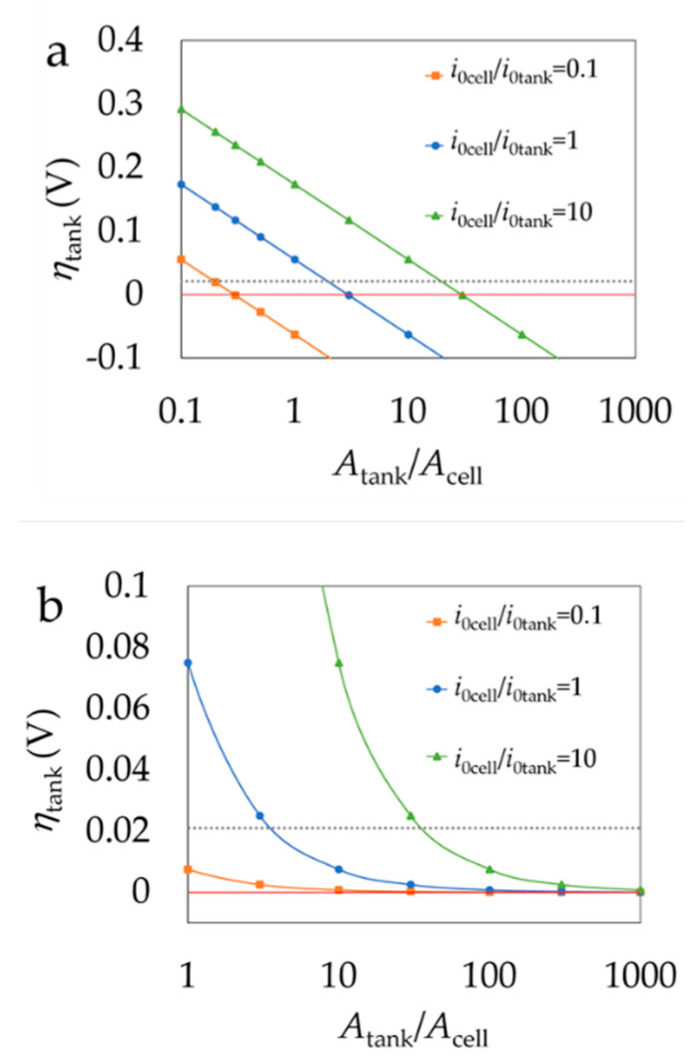
Overpotential of reaction in the tank (*η*_tank_)’s dependence on the ratio of active area of solid beads in the tank (*A*_tank_) and active area of electrode (*A*_cell_) for three cases with different exchange current ratios. The overpotential in the electrode (|*η*_cell_|) is 0.055 V. (**a**) Equation (20). (**b**) Equation (22). Dashed lines mark the 21 mV of overpotential available from the 10% SoC change.

Setting |*η*_cell_| = 0.055 V, overpotential in the tank calculated with Equations (20) and (22) are shown in Figure 10.

From Figure 10 it can be concluded that in case of equal exchange currents (*i*_0cell_/*i*_0tank_ = 1) the active area of solid booster beads should be at least three times the active area of the electrodes. In that case, *η*_tank_ becomes lower than the thermodynamically available overpotential 21 mV. In practice, more surface area is required as some overpotential is also required to drive the intercalation reaction. If the exchange current in the cell is one magnitude larger than the exchange current in the tank (*i*_0cell_/*i*_0tank_ = 10), the active area of solid booster beads should be thirty times larger than the area of the electrodes. Negative values in Figure 10a are because Equation (20) is not valid at low overpotentials. Instead, the behavior of Equation (22) is correct at high overpotentials, where Equation (20) fails. More accurate calculations would require numerical simulations. If the power density of RFB is 0.1 W/cm^2^ and the thickness of carbon felt electrodes is 1 mm, the total volume of electrodes on one side of a 1 kW rated RFB would be 1 dm^3^. Making the estimations that 50% of the tank is filled with solid booster beads, the active area per volume in the tank and cell are equal (*A*_ank_/*V*_tank_ = *A*_cell_/*V*_cell_) and area of cell needs to be thirty times larger than area of electrodes, the total volume of the tank needs to be at least 60 dm^3^. 1 kWh VRFB requires ca. 30 dm^3^ of electrolyte [17], and 4 h battery would have the tank volume of 120 dm^3^. Therefore, these area ranges could be reached with typical systems. Increasing the tank size would increase the total capacity and not affect the power output of the RFB. However, it must be emphasized that mass transfer limitations are not considered for these calculations. In a real system it is essential to minimize mass transfer losses by optimal structure of the electrode and solid booster beads. Theoretically the smaller the active material particles the shorter distance redox active species must travel in the solid particle and the faster the chemical reaction is. However, for system design point of view it must be ensured that the insoluble solid particles stay in the tank and not travel to stack as this might hinder the flow of solution and clog the electrode which could result in elevated resistance of the cell and increased power of the pumps. Therefore, for optimal performance of the RFB the solid beads should be in millimeter scale so they can be easily entrapped in the tank and the mass diffusion losses are minimal.

**Figure 11:**
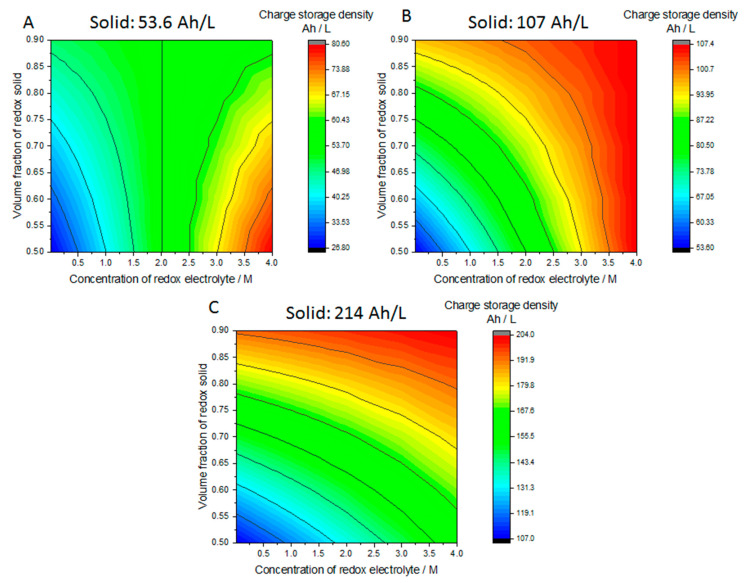
Volumetric charge storage capacity of solid boosted flow battery considering the concentration of the redox electrolyte and the volume fraction of the solid materials. The concentration of the redox active solid is: (**A**) 2 M; (**B**) 4 M; (**C**) 8 M. The volume fraction of additives is considered to be 0, and the number of transferred charge is 1 for all the reactions.

Lastly, we will consider the charge storage capacity of the solid boosted system. Volumetric charge storage capacity of solid boosted flow battery can be estimated as follows:

(24) $$Q/V=(1-\varepsilon_{s}-\varepsilon_{a})n_{l}Fc_{l}+\varepsilon_{s}n_{s}Fc_{s}$$

where *Q* is charge, *V* is the total volume of electrolyte and booster, *ε_s_* is the volume fraction of the redox active solid material, *ε_a_* is the volume fraction of different additives such as the binder and conductive carbon. *F* is the Faradays constant, *n* and *c* are the number of transferred charge and the concentration of the redox active materials, while subscripts l and s refer to liquid and solid phases, respectively. Similar expressions have been used earlier [22]. The unit is C/L, and can be converted to Ah/L by dividing with 3600. Figure 11 plots the charge storage capacity of a flow battery with solid boosters for different redox electrolyte concentrations from 10 mM to 4 M and different volume fractions of redox solids. Three different concentrations for redox active materials, 2 M, 4 M and 8 M are considered. These correspond to charge storage capacity of 54; 107 and 214 Ah/L. For example, FePO_4_ has a charge storage density of 170 mA/g and density of 3.6 g/L, so the active material concentration reaches 22.5 M and the volumetric charge storage capacity 612 Ah/L.

Figure 11 illustrates that high charge storage densities can be reached even with very small concentrations of redox electrolytes. The strong advantage of the redox boosted flow batteries is therefore, that the solubility of the redox electrolyte species does not limit the total charge storage capacity of the system. This significantly enlarges the number of prospective molecules for flow batteries. It is important to remember that up to 80% of the capacity of the solid can be accessible, as illustrated in Figure 5, but this requires very good matching of the SoC curves of the solid and liquid species. On the other hand, concentration of the redox electrolyte defines the power output of the battery, but this decrease in output power can be mitigated by adding more cells to the system.

From an economical point-of-view, inexpensive and stable redox solid materials are required. Compared to aqueous ion batteries, solid boosted flow batteries do not require any current collectors, separators or packing. Instead, active material needs to be formulated to millimeter sized porous beads, and can be just deposited into the tanks of the flow battery. As energy is stored mostly in the solids, utilization of slightly more expensive redox electrolytes could also become feasible. The tanks may need to be redesigned to optimize the reactions, but this cost will not be significant. From a sustainability point-of-view, solid boosted flow batteries should be relatively straightforward to recycle. The electrolyte can be drained, and the solid booster beads can easily be removed from the system.

## 3. Conclusions

Thermodynamical treatment allows for evaluation of the requirements for solid boosted redox flow batteries. 80% capacity utilization of the solid materials can be reached if the redox potentials of the redox electrolyte and the redox solid match well. However, even 50 mV offset results in sharp drop in the capacity utilization of the solid.

Charge transfer between redox electrolyte and redox solid can proceed through different pathways. There can be a direct chemical reaction between the two redox couples, or redox electrolyte can transfer electrons first to a conductive additive. This additive will then shuttle the electrons to the redox active material. In this case, the Fermi level or the potential of the conductive additive can be considered floating. The exact position will be between the Fermi levels of the redox electrolyte and the redox solid, so that both rates of electrolyte reaction and solid reaction are the same.

As the area available for the reactions is much higher in the tank than in the cell, even the small overpotentials due to the changes in the electrolyte SoC are enough to drive the reactions at reasonable rates in the tank. With some assumption, 30 times as much area in the tank should be sufficient to drive the reactions in the tank at the same rate as in the cell.

Solid boosters can significantly enhance the charge storage density of flow batteries, enabling utilization of more expensive redox electrolytes in lower concentrations. This significantly expands the amount of molecules utilizable in practical applications, provided that inexpensive boosters can be found with the right redox potentials. Therefore, solid boosters are a promising technology for large scale stationary energy storage.

## Figures and Tables

**Figure 6:**
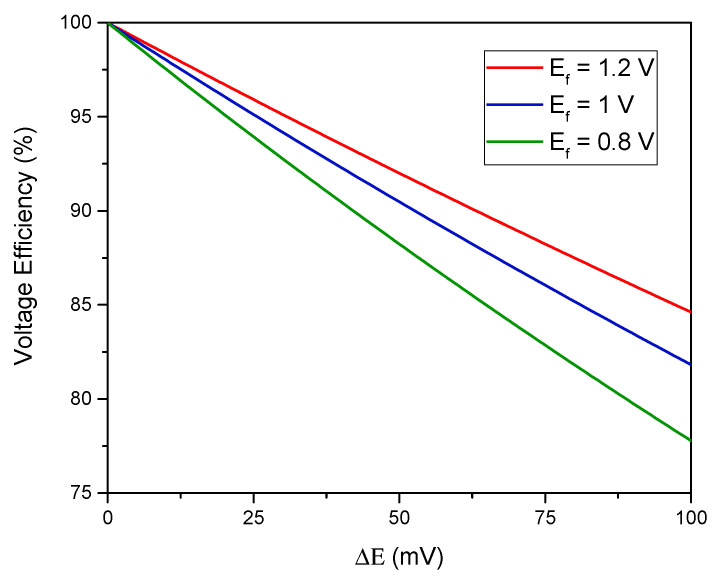
The effect of ΔE on the voltage efficiency.

## Data Availability

Not applicable

## Sample Availability

Samples of the compounds are not available from the authors.
